# Public acceptance of using artificial intelligence-assisted weight management apps in high-income southeast Asian adults with overweight and obesity: a cross-sectional study

**DOI:** 10.3389/fnut.2024.1287156

**Published:** 2024-02-07

**Authors:** Han Shi Jocelyn Chew, Palakorn Achananuparp, Mayank Dalakoti, Nicholas W. S. Chew, Yip Han Chin, Yujia Gao, Bok Yan Jimmy So, Asim Shabbir, Lim Ee Peng, Kee Yuan Ngiam

**Affiliations:** ^1^Alice Lee Centre for Nursing Studies, Yong Loo Lin School of Medicine, National University of Singapore, Singapore, Singapore; ^2^School of Computing and Information Systems, Singapore Management University, Singapore, Singapore; ^3^Department of Cardiology, National University Heart Centre, Singapore, Singapore; ^4^Yong Loo Lin School of Medicine, National University of Singapore, Singapore, Singapore; ^5^Division of Hepatobiliary and Pancreatic Surgery, Department of Surgery, National University Hospital, Singapore, Singapore; ^6^Division of General Surgery (Upper Gastrointestinal Surgery), Department of Surgery, National University Hospital, Singapore, Singapore

**Keywords:** artificial intelligence, obesity, implementation, acceptability, weight management, behavior, UTAUT, perception

## Abstract

**Introduction:**

With in increase in interest to incorporate artificial intelligence (AI) into weight management programs, we aimed to examine user perceptions of AI-based mobile apps for weight management in adults with overweight and obesity.

**Methods:**

280 participants were recruited between May and November 2022. Participants completed a questionnaire on sociodemographic profiles, Unified Theory of Acceptance and Use of Technology 2 (UTAUT2), and Self-Regulation of Eating Behavior Questionnaire. Structural equation modeling was performed using R. Model fit was tested using maximum-likelihood generalized unweighted least squares. Associations between influencing factors were analyzed using correlation and linear regression.

**Results:**

271 participant responses were analyzed, representing participants with a mean age of 31.56 ± 10.75 years, median (interquartile range) BMI, and waist circumference of 27.2 kg/m^2^ (24.2–28.4 kg/m^2^) and 86.4 (80.0–94.0) cm, respectively. In total, 188 (69.4%) participants intended to use AI-assisted weight loss apps. UTAUT2 explained 63.3% of the variance in our intention of the sample to use AI-assisted weight management apps with satisfactory model fit: CMIN/df = 1.932, GFI = 0.966, AGFI = 0.954, NFI = 0.909, CFI = 0.954, RMSEA = 0.059, SRMR = 0.050. Only performance expectancy, hedonic motivation, and the habit of using AI-assisted apps were significant predictors of intention. Comparison with existing literature revealed vast variabilities in the determinants of AI- and non-AI weight loss app acceptability in adults with and without overweight and obesity. UTAUT2 produced a good fit in explaining the acceptability of AI-assisted apps among a multi-ethnic, developed, southeast Asian sample with overweight and obesity.

**Conclusion:**

UTAUT2 model is recommended to guide the development of AI-assisted weight management apps among people with overweight and obesity.

## 1 Introduction

According to the World Health Organization (1), approximately 39 and 13% of the adult population worldwide were living with overweight and obesity, respectively. Among the four million deaths related to having a high body mass index (BMI), more than 66% of them were attributed to cardiovascular diseases among other common chronic diseases, including diabetes mellitus, chronic renal diseases, cancers, and musculoskeletal disorders (2). While pharmacological and surgical interventions have been demonstrated to lead to quick and successful weight reduction, behavior change remains the safest first-line weight management option (3). Several factors have been identified to improve sustained weight loss including adhering to high levels of physical activity, calorie restriction, weight and dietary self-monitoring, self-restraint, confidence, and the low personality trait of novelty seeking (4). However, weight management programs focused on calorie restriction and regular physical exercise are mentally and physically demanding, especially for those with a higher BMI, leading to high non-adherence and attrition rates (5). A 10% increase in adherence would reduce one’s BMI by 2.59 points, suggesting the importance of increasing the acceptability, uptake, and engagement in such programs (6). Various behavior change strategies such as counseling (e.g., motivational interviewing) (7), psychotherapy (e.g., cognitive behavioral therapy) (8), special diet patterns (e.g., time-restricted eating) (9), and exercise regimes (e.g., high-intensity training) (10) have been used to increase one’s motivation and commitment to behavioral change. Various modes of delivery such as face-to-face, web-based, mobile phone apps, and group sessions have also been trialed to improve the effectiveness of various weight loss programs (11, 12). However, the average adherence rate of such programs was reportedly only 60.5%, including those who may have attended every program session but have not necessarily performed the lifestyle change recommended (13).

Alongside the increase in smartphone penetration worldwide, weight loss apps provide users with the convenience of accessing weight loss programs anywhere, at any time. Examples include MyFitnessPal (14), which is essentially a calorie counter to monitor energy intake and expenditure; WW (Weight Watchers) (15), which is an incentive-based points system that encourages users to adhere to their daily meal plans; and Noom (16), which provides health coaching on top of calorie counting. While users of such apps report significant weight loss (17), the first hurdle toward success is the adoption of such programs by the mass public, especially those with overweight and obesity. Common reasons for the non-adoption of such programs include depleting motivation, the lack of satisfactory results, dietary constraints, logistical constraints, low level of supervision, and low provision of social support (13, 18). However, these functions are resource-intensive (e.g., manpower, technical support, and infrastructure) to deploy and maintain.

Artificial intelligence (AI) has been increasingly popular in enhancing the resource efficiency of various activities such as entertainment (e.g., personalized video recommendations on Netflix) and contexts such as smart cities (e.g., energy consumption prediction). AI is capable of offloading the demand for manpower through human–AI interventions, which imitates human intelligence and communications to perform human tasks faster, more accurately, and more efficiently (19, 20). However, the effectiveness of AI-assisted weight loss apps remains unclear and is largely contingent upon the user’s engagement with the app’s contents. Several studies have examined the acceptability of weight loss apps, but few studies have considered those with AI enhancements, the influencing factors of acceptability in a Southeast Asian multi-ethnic population (21–23). For example, one study on college students reported that out of the seven influencing factors of usage intention included in Unified Theory of Acceptance and Use of Technology 2 (UTAUT2), only performance expectancy, hedonic motivations, price value, and habit significantly predicted app acceptability (24) On the contrary, a study on people with overweight and obesity showed that only performance expectancy, effort expectancy, and social influence influenced app acceptability (25).

While studies have shown positive perceptions of the use of AI in healthcare such as increased availability, user-friendliness, and cost-efficiency, common barriers include concerns about data privacy, credibility, patient safety, and technological maturity (26). With the increasing use of AI in weight loss apps, it is timely to assess the needs, preferences, and influencing factors of AI and non-AI smartphone-based weight management apps in a multi-ethnic context like Singapore. Therefore, we aimed to examine the user perceptions of artificial intelligence (AI)-based mobile apps for weight management in people with overweight and obesity. Broadly, this study would also indicate the technological readiness of Singaporeans who are overweight and obese to adopt AI-based technology.

## 2 Materials and methods

A cross-sectional study is reported as part of a sequential explanatory study. This study was approved by the National Healthcare Group (NHG) Domain Specific Review Board (DSRB) Ethics Review Board (ref: 2021/00834) and registered with ClinicalTrials.gov (ref. NCT05257239). The results are reported according to the STrengthening the Reporting of OBservational studies in Epidemiology (STROBE) checklist for cross-sectional studies (Supplementary Table S1).

### 2.1 Participants and procedure

Participants were recruited from the public and a specialist outpatient weight management center via social media advertisements and face-to-face engagements, respectively, from May to November 2022. Participants were included if they were above 21 years old, had a BMI ≥23 kg/m^2^, and understood the English language. There were no exclusion criteria to maximize the representation of the findings of the general Singaporean population with overweight and obesity. Based on a 28.8% prevalence of overweight in Singapore, a total of 261 participants were needed to represent the population at 80% power with a 5.5% margin of error. To be conservative, 280 participants were recruited to account for the potential voiding of responses.

### 2.2 Data collection

Participants were required to complete an online survey that included questions on their sociodemographic profile, the UTAUT2 questionnaire, and the Self-Regulation of Eating Behavior Questionnaire (SREBQ). The median time taken to complete the questionnaires with 51 items was 7.1 min (interquartile range: 5.5–10.8 min). Participants were also asked whether they had experience with using a weight loss app and to specify the app name if applicable. As mentioned in another article, participants were asked to describe their understanding of the difference between AI and non-AI apps. The interviewer then shared briefly that “AI is a semi-autonomous machine or system that can enhance the speed and efficacy of routine tasks through machine learning” (under review). Examples were also provided.

#### 2.2.1 Sociodemographic profile

Participants completed a survey on their age, sex, marital status, race, religion, highest education level, employment, and *per capita* household income. Height, weight, and waist circumference were self-reported. To lower the risk of participants misreporting their weight status, our study team members verified visually through a virtual face-to-face meeting during consent taking.

#### 2.2.2 UTAUT2

The UTAUT2 is a 21-item questionnaire judged on a 7-point scale (1 = strongly disagree, 7 = strongly agree) that has been widely used to assess one’s behavioral intention to adopt technological applications (27). It comprises seven constructs namely performance expectancy (four items), effort expectancy (four items), social influence (three items), facilitating conditions (four items), hedonic motivation (three items), price value (three items), and habit (four items). The UTAUT2 was adapted for AI-based weight loss mobile apps.

#### 2.2.3 SREBQ

The SREBQ is a five-item questionnaire measured on a 5-point scale (1 = Never; 2 = Rarely; 3 Sometimes; 4 = Frequently; 5 = Always) (α = 0.75) (28). An example of an item is “I’m good at resisting tempting food.” Of the five items, three items were to be reverse coded of which a mean score of <2.8 indicates low self-regulation, 2.8–3.6 indicates moderate self-regulation, and > 3.6 indicates high self-regulation. Additionally, participants were required to indicate which of a list of food items was most enticing to them. SREBQ has been validated with a strong positive correlation with general measures of self-regulation and negative correlations with food responsiveness and emotional overeating (28).

### 2.3 Data analysis

R software (lavaan package) was used to conduct structural equation modeling (SEM). SEM is a statistical method that combines factor analysis and multiple regression analysis to analyze the relationships between observed and latent variables. A two-stage SEM approach was used by first examining the measurement model for its reliability and validity and then estimating the goodness of fit of the structural model. Model fits were tested using maximum-likelihood generalized unweighted least squares. Descriptive statistics were used to report the central tendencies of the measured variables. Correlation analysis and linear regression were conducted to describe the association between factors.

## 3 Results

### 3.1 Participant characteristics

Two hundred eighty participants were recruited of which nine responses were voided as the participants were of a normal weight status (i.e., BMI < 23 kg/m^2^). The sociodemographic profile of the 271 participants included in our analysis is shown in Table 1. The mean age ± standard deviation of the participants was 31.56 ± 10.75 years old, and there was a proportionate mix of sexes (50.4% females and 49.6% males). The median (interquartile range; IQR) BMI and waist circumference were 27.22 kg/m^2^ (24.2–28.4 kg/m^2^) and 86.36 (80.0–94.0) cm, respectively. Most of the participants were of Chinese race (83.0%), single (75.2%), had a university-level education (66.3%), had a *per capita* income of 5,001–10,000 Singaporean dollars, and were working full-time. Of the 41 participants who named the weight loss apps used, most had used the following weight loss apps: MyFitnessPal (n = 31), eTRIP (n = 9), Feelfit (n = 4), Lifesum (n = 3), LoseIt! (n = 3), nbuddy (n = 3), Noom (n = 3), Fitbit (n = 2), Healthy365 (n = 2), Intermittent fasting (n = 2), and Samsung Health (n = 2).

**Table 1:** Sociodemographic profile of the 271 participants with overweight and obesity.

Sociodemographic characteristics	Mean ± SD/count (%)
Age	31.56 ± 10.73
Sex	
Female	137 (50.6)
Male	134 (49.4)
Marital status	
Single	204 (75.3)
Married	60 (22.1)
Divorced	7 (2.6)
Race	
Chinese	225 (83.0)
Indian	31 (11.4)
Malay	10 (3.7)
Others	5 (1.8)
Religion	
Buddhism	91 (33.6)
Christianism	67 (24.7)
Hinduism	23 (8.5)
Islam	18 (6.6)
Freethinker	59 (21.8)
Others	13 (4.8)
Highest educational qualification	
Primary	1 (0.4)
Secondary	13 (4.8)
Pre-university	77 (28.4)
University	180 (66.4)
*Per capita* income, SGD	
<1,000	18 (6.6)
1,000-3,000	69 (25.5)
3,001-5,000	66 (24.4)
5,001-10,000	87 (32.1)
>10,000	31 (11.4)
Employment status	
Part-time	42 (15.6)
Full-time	189 (69.7)
Retired	3 (1.1)
Student	25 (9.2)
Unemployed	12 (4.4)
BMI, kg/cm^2^; median (IQR)	25.9 (24.2–28.4)
At risk (23–24.9 kg/cm^2^)	98 (36.2)
Obesity class I (25–29.9 kg/cm^2^)	131 (48.3)
Obesity class II (≥30 kg/cm^2^)	42 (15.5)
Waist circumference, cm; median (IQR)	86.4 (80.0–94.0)
High (male ≥90 cm; female ≥80 cm)	150 (55.4)
Prior experience with using weight loss apps	200 (74.1)

SD, standard deviation; SGD, Singapore dollars; IQR, interquartile range; median and IQR are reported for variables with skewness between −2 to +2 and kurtosis between −7 to +7.

### 3.2 Descriptive statistics of UTAUT2 items

The descriptive statistic of each questionnaire is detailed in Table 2; 188 (69.4%) participants intended (mean intention score > 4) to use AI-assisted weight loss apps. The mean UTAUT2 item responses were positive (mean score > 4 which represents the response “Unclear”) for all constructs except for the habit of using AI-assisted weight loss apps (3.43 ± 1.56). Interestingly, while 86.3% of the participants expressed the intention to cut down on tempting food consumption, only 13.7% responded to eat healthily. The top three most tempting foods were sweets (56.7%), chocolate (55.2%), and ice cream (50.7%); 24.4 and 19.3% of the participants were at risk of having anxiety and depression symptoms. The intention to use AI-assisted weight management apps was associated with age, anxiety risk, the intention to have a healthy diet, and all seven constructs in UTAUT2, which accounted for 61.6% of the variation in intention to use AI-assisted weight management apps. Self-regulation, depression risk, BMI, and waist circumference were not associated with the use of AI-assisted weight management apps (Supplementary Table S2).

**Table 2:** Descriptive statistics for the questionnaire items responses of the 271 participants.

Sociodemographic characteristics	Mean ± SD/count (%)
UTAUT2	
Intention	4.90 ± 1.38
Performance expectancy	4.77 ± 1.21
Effort expectancy	5.20 ± 1.14
Social influence	3.86 ± 1.48
Facilitating conditions	5.38 ± 1.08
Hedonic motivation	4.68 ± 1.33
Price value	4.24 ± 1.08
Habit	3.43 ± 1.56
SREBQ	2.94 ± 0.56
Low	86 (31.7)
Moderate	166 (61.3)
High	19 (7.0)
Intention to cut down on tempting food consumption	233 (86.3)
Intention to eating healthily	37 (13.7)
Tempting foods	
Sweets	154 (56.8)
Chocolate	150 (55.3)
Ice cream	137 (50.7)
Others	116 (43.0)
Biscuits	111 (41.1)
Cake	111 (41.1)
Fizzy drinks	104 (38.5)
Fried foods	104 (38.7)
Crisps	88 (32.5)
Chips	74 (27.3)
Bread/toast	59 (21.8)
Pastries	47 (17.3)
Popcorn	40 (14.8)
Pizza	38 (14.0)
Nil	7 (2.6)
GAD-2	1.7 ± 1.67
Potentially at risk (≥3)	67 (24.7)
PHQ-2	1.5 ± 1.53
Potentially at risk (≥3)	52 (19.3)

UTAUT2, Unified Theory of Acceptance and Use of Technology 2 adapted for the use of AI-assisted weight loss apps; SREBQ, Self-Regulation of Eating Behaviour Questionnaire; GAD-2, Generalized Anxiety Disorder 2-item; PHQ-2, Patient Health Questionnaire-2.

### 3.3 Measurement model

All items fulfilled the assumption of normality for SEM (skewness <2; kurtosis <7) (29). Construct validity of the measurement model with nine latent constructs (PE, EE, SI, FC, HM, HT, PV, BI, and SR) was first examined using confirmatory factor analysis, followed by convergent and discriminant validity. Construct reliability was examined using Cronbach’s alpha (α), composite reliability (CR), average variance extracted (AVE), maximum shared variance (MSV), and average shared variance (ASV).

#### 3.3.1 Model fit

Model fit was evaluated via the model chi-square comparative fit index (CFI) (30), root mean square error of approximation (RMSEA), and standardized root mean square residual (SRMR) (31). As shown in Table 3, all fit indices of the first measurement model were acceptable. On inspection of the standard regression weights (factor loading) (36), we realized that SR4 (factor loading (FI): −0.044) and SR5 (FI: 0.432) had low factor loading (<0.50) and hence were removed.

**Table 3:** Fit indices of measurement scale.

Fit indices	Measurement model 1	Measurement model 2	Structural model	Recommended values (29)
CMIN/df	1.857	1.910	1.932	<3 (32)
GFI	0.971	0.967	0.966	≥ 0.90 (33)
AGFI	0.962	0.967	0.954	≥ 0.80 (34)
NFI	0.901	0.911	0.909	≥ 0.90 (35)
CFI	0.952	0.955	0.954	≥ 0.90 (30)
RMSEA	0.056	0.058	0.059	≤ 0.08
SRMR	0.059	0.049	0.050	≤ 0.08

CMIN/DF, Chi-square divided by degree of freedom; GFI, goodness-of-fit Index; AGFI, adjusted goodness-of-fit index; NFI, normed fit index; CFI, comparative fit index; RMSEA, root mean square error of approximation.

#### 3.3.2 Construct validity and reliability

In terms of convergent validity, all retained items had a standardized regression weight ranging from 0.608 to 0.974, indicating acceptable to excellent factor loading on each latent variable (≥0.4 to ≥0.7) (34). The factor loading of all items on their respective latent variables was also statistically significant (*p* < 0.001). In terms of construct reliability, all retained items had a satisfactory α > 0.70, AVE >0.50, and CR >0.70 (Table 4) (36). In terms of discriminant validity, all items had an AVE larger than MSV and ASV (Table 4) (36). The highest correlation coefficient among latent constructs is 0.768; hence, the MSV is 0.590.

**Table 4:** Cronbach’s alpha (α), composite reliability (CR), average variance extracted (AVE), maximum shared variance (MSV), and average shared variance (ASV).

Latent constructs	ɑ	CR	AVE	ASV
Intention	0.938	0.942	0.843	0.283
Performance expectancy	0.926	0.928	0.762	0.325
Effort expectancy	0.927	0.928	0.762	0.281
Social influence	0.962	0.962	0.894	0.231
Facilitating conditions	0.851	0.859	0.606	0.137
Hedonic modification	0.962	0.963	0.896	0.289
Price value	0.920	0.894	0.794	0.187
Habit	0.942	0.942	0.803	0.255
Self-regulation of eating habits	0.767	0.781	0.547	0.012

#### 3.3.3 Common method bias

To avoid the risk of common method bias, the nine constructs with 23 scale items were assessed using Harman’s single-factor method (37). Factor analysis with principal axis factoring and no rotation was conducted. No common method bias was found as only 1 factor emerged to account for 40.0% of the variance, which is less than the threshold of 50% (38). The next factor with the highest eigenvalues explained only 9.86% of the variance.

### 3.4 Structural model

**Figure 1:**
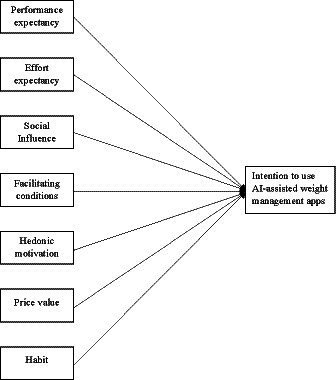
Illustration of the hypothesized UTAUT2 model.

The structural model shown in Figure 1 was tested to have satisfactory fit statistics (Table 3) of which PE, EE, SI, FC, HM, PV, and HT explained 63.3% of the variance in INT. Self-regulation was taken out from the model to improve the model fit. The results of the hypothesized pathways indicated that the intention to use AI-assisted weight management apps was directly predicted by PE, HM, and HT but not EE, SI, FC, and PV (Supplementary Table S3).

## 4 Discussion

To the best of our knowledge, this is the first study to examine the public acceptance and intention to use AI-assisted weight management apps in Singapore, a high-income multi-ethnic country that was ranked fourth in the International Institute for Management Development (IMD) World Digital Competitiveness Ranking 2022, following Denmark, the United States, and Sweden (first) in increasing rank order (39). Recognizing the important role of AI in the future, Singapore also prioritizes AI research and launched a national AI strategy in 2019 (40).

Generally, the seven UTAUT2 constructs explained 63.3% of the variance in our sample’s intention to use AI-assisted weight management apps, similar to another study on 439 German users with overweight and obesity where the UTAUT model explained 60.0% of the variance (25). However, this study was not specific to AI-assisted apps, where the incorporation of AI into healthcare devices and services has always been contended for its potential to breach data privacy and trust issues (26). However, replacing the price value construct with trust and privacy concerns only explained 20% of the variance in the intention to use non-AI-specific lifestyle and therapy apps (41). Although this could be due to a larger predictive strength of price value than trust and privacy concerns on intention, this is more likely due to testing the UTAUT model on a general population as compared to a targeted population in the former two studies on adults with overweight and obesity. In terms of adapted UTAUT models, one study on a Taiwanese sample reported that the UTAUT2 constructs alongside personal innovativeness and network externality explained 75.5% of the AI-assisted weight loss app acceptability among the general public (42). These findings suggest that the application of UTAUT2 in developing and evaluating technology-related healthcare services may not be generalizable across populations and the effectiveness of such interventions should be customized to the needs of certain populations. The integration of the qualitative findings from the larger study is reported elsewhere.

Interestingly, only performance expectancy, hedonic motivation, and the habit of using AI-assisted apps were significant predictors of intention. Our literature search revealed vast variabilities in the determinants of weight loss app acceptability. Concurring with our findings, the most common determinants were habit and performance expectancy, which were identified by six studies in both the general and overweight/obese populations (17, 24, 42–45). However, three studies (17, 43, 45) also found effort expectancy and social influence to be significant predictors of behavioral intention while another study on the general population identified performance expectancy, effort expectancy, and social influence as significant determinants (25). Two studies (17, 24) found hedonic motivation and price value to be significant predictors of behavioral intention in the general population, whereas facilitating condition was identified as one of the key predictors of behavioral intention in a sample attending cardiac rehabilitation (43). Such variability could be due to the different determinants that influence AI-assisted and non-AI-assisted weight loss apps differently. Our findings suggest that users were willing to use AI-assisted apps if they perceived a high chance of weight loss success and used them repeatedly, regardless of the effort, price, resources, and social influence present. As both Singapore and Taiwan are developed countries with relative financial stability (42), users may be less sensitive to the price, effort, and resources available. Due to the relative novelty of AI-assisted apps, the absence of social influence as a significant predictor could also be due to the lack of experience and knowledge about it as compared to general weight loss apps as indicated by the low scores on the habit of use (3.43 ± 1.56).

Interestingly, while 86.3% of the participants expressed the intention to cut down on tempting food consumption, only 13.7% responded to eating healthily. This presents an irony where users may intend to reduce the intake of only certain junk foods and not compensate for this with healthy food (e.g., fruits and vegetables) intake (46). The top three most tempting foods were sweets (56.7%), chocolate (55.2%), and ice cream (50.7%), which are calorie-dense obesogenic foods that highlight the need for interventions to reduce the consumption of such foods. Interestingly, self-regulation, depression risk, BMI, and waist circumference were not associated with intention to use AI-assisted weight management apps, potentially because these constructs are more distal than the UTAUT2 constructs in terms of their influence over the intention. Moreover, although 24.4% and 19.3% of the participants were at risk of having anxiety and depression symptoms, these risks were not significantly associated with BMI or waist circumference, which contradicts existing studies (47). This could be because the association only becomes significant when the anxiety and depression risk is confirmed with a diagnosis or because these psychiatric disorders are such a taboo in an Asian country that the prevalence was underreported in this study (48).

There were several limitations to this study. First, although the ethnic sample composition is representative of the local population, the sample is still relatively small as compared to census data. This alongside the use of self-reports would limit the generalizability of our findings. Second, although we explained the differences between AI and non-AI weight loss apps, participants may not fully appreciate the differences and hence lower the accuracy of our findings. Future studies could consider conducting a short test to ensure that participants fully understand the differences between AI and non-AI applications. Longitudinal studies could also be conducted to observe changes in user perceptions over time, and experimental studies could be conducted to evaluate the effectiveness of specific app features. This would inform the development of weight loss apps to be comprehensive yet limited redundancy. As only performance expectancy, hedonic motivation, and the habit of using AI-assisted apps were significant predictors of intention, weight management apps could be designed to track and visualize one’s weight loss progress, and intrinsic motivation, and promote repetition of healthy eating habits. Large language models (LLMs) could also be used to explore app feedback and social media comments to further understand the various predictors of user adoption. LLMs could also be used to provide personalized plans and feedback to enhance the effectiveness and user experience of AI-assisted weight management apps (19).

## 5 Conclusion

The UTAUT2 was found to produce a good fit in explaining the acceptability of AI-assisted apps among a multi-ethnic, developed, southeast Asian sample with overweight and obesity. Therefore, the UTAUT2 model is recommended to guide the development of AI-assisted weight management apps among people with overweight and obesity.

## Data availability statement

The raw data supporting the conclusions of this article will be made available by the authors, without undue reservation.

## Ethics statement

The studies involving humans were approved by National Healthcare Group Domain Specific Review Board. The studies were conducted in accordance with the local legislation and institutional requirements. The participants provided their written informed consent to participate in this study.

## Author contributions

HC: Conceptualization, Data curation, Formal analysis, Funding acquisition, Investigation, Methodology, Project administration, Resources, Software, Supervision, Validation, Visualization, Writing – original draft, Writing – review & editing. PA: Conceptualization, Writing – original draft, Writing – review & editing. MD: Writing – review & editing, Validation. NC: Validation, Writing – review & editing. YC: Validation, Writing – review & editing. YG: Validation, Writing – review & editing. BJ: Validation, Writing – review & editing. AS: Validation, Writing – review & editing. LP: Validation, Writing – review & editing. KN: Validation, Writing – review & editing.
